# A Retroperitoneal Isolated Enteric Duplication Cyst Mimicking a Teratoma: A Case Report and Literature Review

**DOI:** 10.1155/2016/6976137

**Published:** 2016-12-19

**Authors:** Daichi Momosaka, Yasuhiro Ushijima, Akihiro Nishie, Yoshiki Asayama, Kousei Ishigami, Yukihisa Takayama, Daisuke Okamoto, Nobuhiro Fujita, Tetsuo Ikeda, Keiichiro Uchida, Masaaki Sugimoto, Kenichi Kohashi, Hiroshi Honda

**Affiliations:** ^1^Department of Clinical Radiology, Graduate School of Medical Sciences, Kyushu University, 3-1-1 Maidashi, Higashi-ku, Fukuoka 812-8582, Japan; ^2^Department of Radiology Informatics and Network, Graduate School of Medical Sciences, Kyushu University, 3-1-1 Maidashi, Higashi-ku, Fukuoka 812-8582, Japan; ^3^Center for Integration of Advanced Medicine and Innovative Technology, Graduate School of Medical Sciences, Kyushu University, 3-1-1 Maidashi, Higashi-ku, Fukuoka 812-8582, Japan; ^4^Department of Medicine and Bioregulatory Science, Graduate School of Medical Sciences, Kyushu University, 3-1-1 Maidashi, Higashi-ku, Fukuoka 812-8582, Japan; ^5^Department of Anatomic Pathology, Graduate School of Medical Sciences, Kyushu University, 3-1-1 Maidashi, Higashi-ku, Fukuoka 812-8582, Japan

## Abstract

Enteric duplication cysts lacking anatomic association with the gastrointestinal tract are called isolated enteric duplication cysts (IEDCs). We present an atypical case of a retroperitoneal IEDC with a tortuous tubular complex shape that enfolded the surrounding retroperitoneal fat and mimicked a retroperitoneal teratoma. Multiplanar reconstruction images should be used to evaluate such a lesion correctly. A tortuous tubular complex shape could be a key finding to differentiate from other retroperitoneal cysts.

## 1. Introduction

Enteric duplication cysts (EDCs) are uncommon congenital anomalies that can be found anywhere along the alimentary tract from the tongue to the anus [1–4]. Essentially they are located in or immediately adjacent to some part of the alimentary tract wall [1]. Histologically, EDCs have a well-developed coat of smooth muscle and an epithelial lining that represents some portion of the intestinal tract mucosa and contain various concentrations of mucus [1]. The incidence rate of EDCs is 1 in every 4000 to 5000 live births [5]. Although the majority of cases are detected in infants, they can be found in patients of any age [6]. Some cases lack anatomic association with the normal gastrointestinal tract, and they are called isolated enteric duplication cysts (IEDCs) [7]. Prenatal vascular accidents, torsion, and heterotopic tumors may be considered the etiology of IEDCs [8]. This type of tumor has been reported in locations including the tongue [1, 2], pleural space [1], liver [9, 10], pancreas [1, 11], biliary tree [2, 11, 12], and retroperitoneum. Only 17 cases of retroperitoneal IEDCs are found in the literature [8, 13–27]. Most cases have a unilocular or multilocular shape.

Herein, we report a case of a retroperitoneal IEDC that formed a curious shape. This mass was misdiagnosed to include a fat component and was difficult to discriminate from a teratoma. We also discuss the radiological findings useful to a correct diagnosis of retroperitoneal IEDC.

## 2. Case Presentation

**Figure 1:**
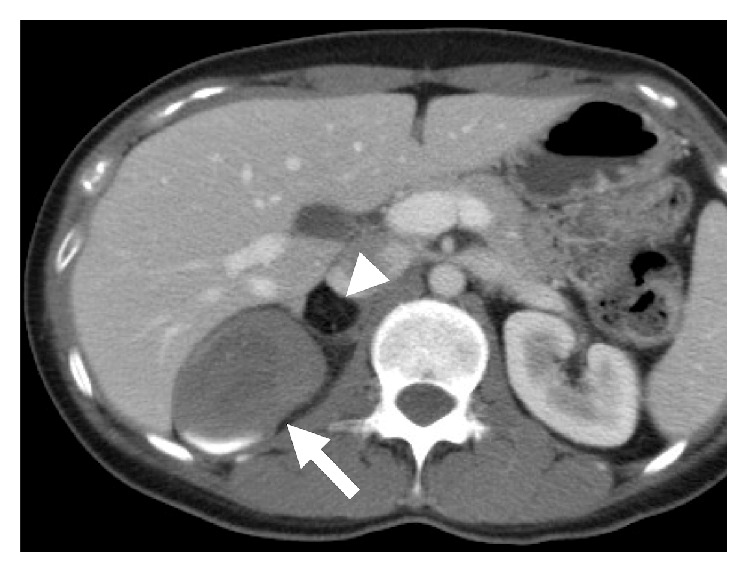
Axial contrast-enhanced CT scan. The mass consisted of a cystic component (arrow) and a nodular fatty component (arrowhead) at the right adrenal region. A high-density fluid-fluid level was also seen in the cystic component. No enhancing component was observed.

**Figure 2:**
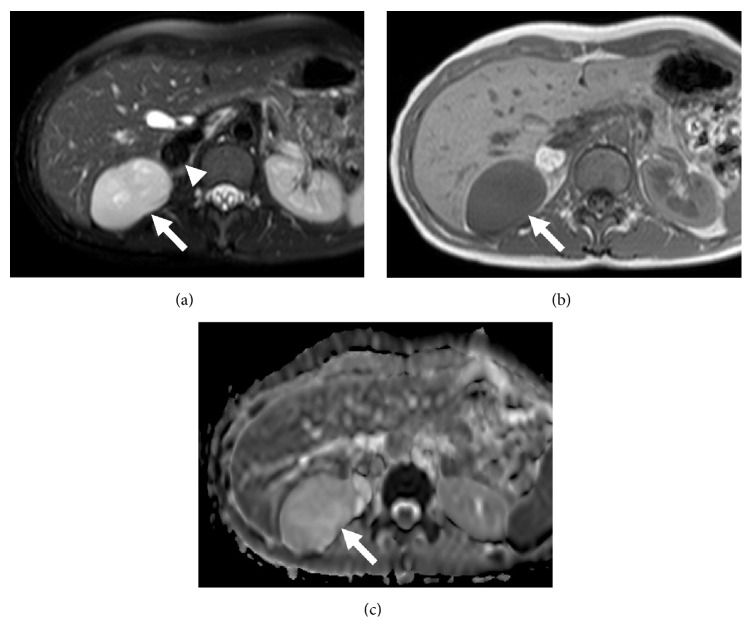
Axial MRI. (a) Fat-suppressed T2-weighted image showed heterogeneous high intensity inside the cystic component of the mass (arrow). The fatty component showed low intensity (arrowhead). (b) T1-weighted image showed relative homogeneous isointensity inside the cystic component of the mass (arrow). (c) ADC map showed slightly restricted diffusion compared with cerebrospinal fluid. Its ADC value was 2.0 × 10^−3^ mm^2^/sec.

**Figure 3:**
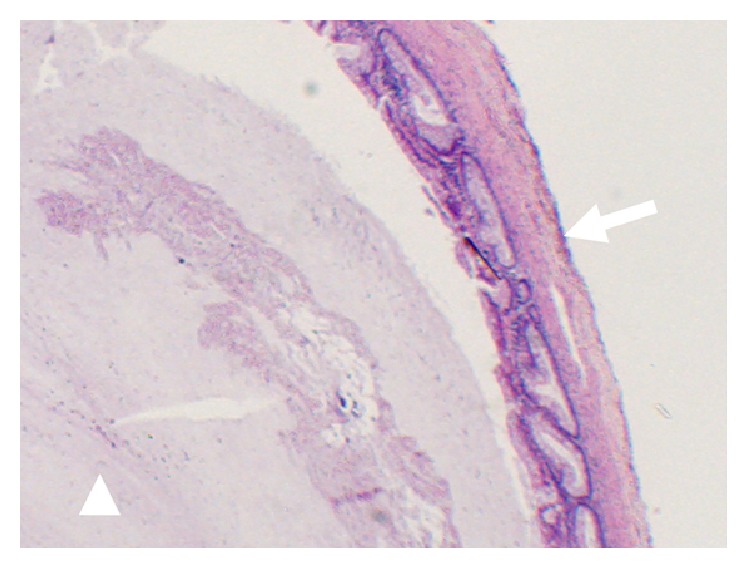
Microphotographs of the cystic wall. The wall consisted of large intestinal mucosa, submucosa, muscle layers, and serosa (arrow). The cyst was filled with viscous mucus (arrowhead). A cellular component was not seen (high magnification, hematoxylin and eosin staining).

**Figure 4:**
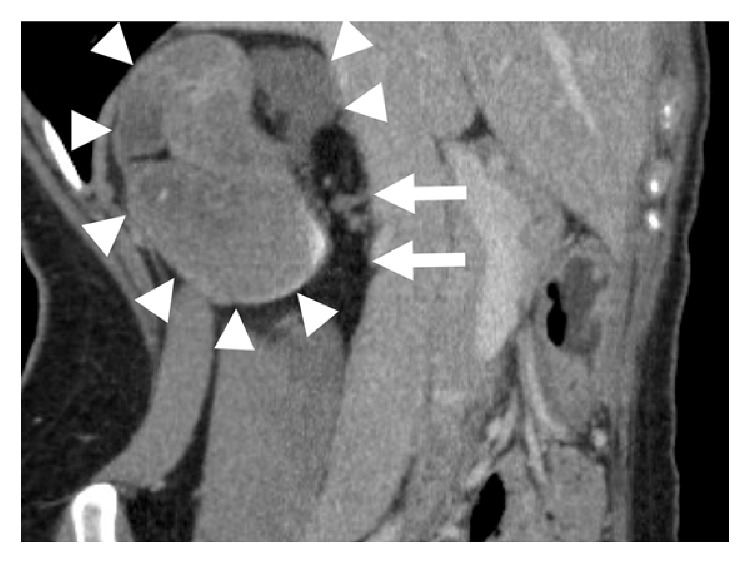
Oblique MPR images of contrast-enhanced CT scan. A tortuous tubular complex shape of the mass (arrowheads) was clearly observed. The nodular fatty component of the mass continued to the normal retroperitoneal fat (arrows).

A 35-year-old woman visited our institution with an abdominal mass detected on abdominal ultrasound. The patient had no history of parity, drug use, or surgical intervention. On contrast-enhanced computed tomography (CT), a mass with a distorted shape and a diameter of 7.5 cm occupied the region between the right lobe of the liver and the right adrenal gland. The mass, which consisted of nodular fatty components, was well-circumscribed without contrast enhancement. A high-density fluid-fluid level was also seen (Figure 1). On magnetic resonance imaging (MRI), the cystic component showed heterogeneous high intensity on fat-suppressed T2-weighted images (Figure 2(a)) and relatively homogeneous isointensity compared to the muscle on T1-weighted images (Figure 2(b)). On chemical shift images, microscopic fat was not observed in the cystic component. On DWI, the cystic component showed slightly high intensity and its ADC value was 2.0 × 10^−3^ mm^2^/sec, suggesting slightly restricted diffusion compared with the cerebrospinal fluid (Figure 2(c)). A nodular fatty component was again identified on MRI (Figures 2(a) and 2(b)). Based on the presence of a fatty component and possibly calcification or iodine inside the mass, our preoperative diagnosis was retroperitoneal teratoma. The patient underwent laparoscopic surgical intervention. Macroscopically, the mass was a tortuous tubular cyst. The nodular fatty component indicated on preoperative images was not a part of the mass but rather a part of the normal retroperitoneal fat that the complex cyst enfolded. The mass was separated from the colon and the right adrenal gland. Microscopically, the wall of the cyst consisted of well-developed smooth muscle and an epithelial lining representing the large-intestine mucosa. Its content was viscous mucus (Figure 3). The final diagnosis was retroperitoneal IEDC. Retrospective multiplanar reconstruction (MPR) oblique images revealed the appearance of a tortuous tubular cyst clearly and showed that the nodular fat-density component of the mass was continuous with the normal retroperitoneal fat (Figure 4).

## 3. Discussion

EDCs are congenital enteric malformations with a cystic appearance, a well-developed coat of smooth muscle, and an epithelial lining representing some portion of the intestinal tract mucosa [28]. On CT and MRI, EDCs are well-circumscribed fluid-filled cysts with a slightly enhanced thin wall, which is located in or adjacent to the normal gastrointestinal wall. The density and intensity of the intracystic fluid can vary depending upon mucous concentration, viscosity, and the existence of intermixed hemorrhage [29, 30]. In our case, the CT and MRI findings on the internal characteristics of the cystic component are consistent with those of the previous reports on EDCs.

The shape of the IEDC in our case is noteworthy. Only 17 cases of retroperitoneal IEDCs have been reported [8, 13–27]. These cases are summarized in Table 1. Retroperitoneal IEDCs demonstrated unilocular (75%) or multilocular to multilobulated (25%) shapes. The present case is the first report of an IEDC with a tortuous tubular complex shape. However, in EDCs that have continuity with the wall of the normal alimentary tract, the shape can be either spherical (80%) or tubular (20%) [31]. That is, 20% of EDCs are tubular. It would be reasonable that IEDCs can also be tubular. To the best of our knowledge, this is the first reported case of a retroperitoneal cyst with such a shape [32].

Another point to discuss is that the mass enfolded surrounding retroperitoneal fat and mimicked a fat-containing tumor. A misdiagnosis resulting from that resemblance may derive from the tortuous tubular complex shape described above. Retrospectively, however, MPR images were useful for differentiating the IEDC from retroperitoneal teratoma because the nodular fat-density component of the mass was continuous with the normal retroperitoneal fat. In addition, MPR images also clearly revealed a tortuous tubular cyst. This image reconstruction technique is of great value for grasping the three-dimensional anatomy of a lesion [33]. For retroperitoneal masses, surgery is basically performed, although ultrasound-guided aspiration and ethanol sclerotherapy can be sometimes performed instead [34]. MPR images can enable surgeons to choose more appropriate operative methods.

In conclusion, a retroperitoneal IEDC can show a tortuous tubular shape and enfold surrounding retroperitoneal fat due to its complex shape. MPR images should be used to evaluate such a lesion correctly. A tortuous tubular complex shape could be a key finding to differentiate from other retroperitoneal cysts.

## Figures and Tables

**Table 1:** Retroperitoneal IEDCs.

Case number	Age/sex	Laterality	Size (cm)	Ectopic gastric mucosa	Ectopic pancreatic mucosa	Shape	Location	Ref.
1	19 y/F	L	11	+	−	Simple	Nearby pancreas	[13]
2	79 y/F	L	5	−	−	Simple	Left adrenal area	[14]
3	34 y/F	R	10	−	−	Simple	Nearby duodenum	[15]
4	19 y/F	L	13	−	−	Multilobular	ND	[16]
5	35 y/F	L	5.5	+	−	Polycystic	Left adrenal area	[17]
6	31 y/F	M	5	−	−	Simple	ND	[18]
7	1 wk/ND	L	3.5	ND	ND	Simple	ND	[19]
8	6 mo/M	R	10	ND	ND	Simple	ND	[19]
9	17 y/M	L	8.6	+	+	Simple	Left adrenal area	[20]
10	27 d/M	L	3	+	−	Simple	Nearby pancreas	[21]
11	28 y/F	L	ND	+	−	Simple	Nearby left kidney	[22]
12	9 d/M	M	5	+	−	Simple	Nearby pancreas	[23]
13	ND/F	Bil	4	−	−	Dumbbell	ND	[24]
14	7 mo/F	R	2	+	−	Polycystic	Right adrenal area	[25]
15	10 mo/F	R	3.8	+	−	Simple	Left adrenal area	[26]
16	9 mo/M	R	ND	+	ND	ND	Nearby right kidney	[27]
17	2 d/M	R	6	−	−	Simple	Nearby extrahepatic bile duct	[8]

IEDCs: isolated enteric duplication cysts, ND: not described, M: male, F: female, Bil: bilateral, R: right, L: left, M: middle, y: years, mo: months, wk: weeks, and d: days.
